# *KRAS* ‐G12C: The neglected biomarker to detect patients with *MUTYH* ‐associated polyposis

**DOI:** 10.1002/ijc.70281

**Published:** 2025-12-05

**Authors:** Ana Beatriz Deleame Medeiros, Gabriel Oliveira dos Santos, José Claudio Casali‐da‐Rocha, Samuel Aguiar Junior, Virgilio Souza e Silva, Gustavo Nóriz Berardinelli, Augusto Perazzolo Antoniazzi, Rui Manuel Reis, Dirce Maria Carraro, Giovana Tardin Torrezan

**Affiliations:** ^1^ Clinical and Functional Genomics, A. C. Camargo Cancer Center São Paulo Brazil; ^2^ Department of Anatomic Pathology A. C. Camargo Cancer Center São Paulo Brazil; ^3^ Oncogenetics Department A. C. Camargo Cancer Center São Paulo Brazil; ^4^ Colorectal Tumors Reference Center, A. C. Camargo Cancer Center São Paulo Brazil; ^5^ Molecular Diagnostic Laboratory, Barretos Cancer Hospital Barretos Brazil; ^6^ Oncogenetics Department Barretos Cancer Hospital Barretos Brazil; ^7^ Molecular Oncology Research Center, Barretos Cancer Hospital Barretos Brazil; ^8^ Life and Health Sciences Research Institute (ICVS), Medical School, University of Minho Braga Portugal; ^9^ National Institute of Science and Technology in Oncogenomics and Therapeutic Innovation São Paulo Brazil

**Keywords:** colorectal cancer, germline pathogenic variants, *KRAS*‐G12C, *MUTYH*‐associated polyposis

## Abstract

*MUTYH*‐associated polyposis (MAP) is an underdiagnosed recessive syndrome that predisposes individuals to colorectal cancer (CRC) and exhibits phenotypic variability. Biallelic *MUTYH* inactivation leads to a somatic mutational signature with frequent *KRAS*‐G12C mutations; however, despite being proposed as a marker for MAP, germline *MUTYH* testing in these patients remains limited. We assessed the utility of screening germline pathogenic variants (GPVs) in *MUTYH* among CRC cases with *KRAS*‐G12C. A cohort of 220 *KRAS*‐G12C CRC patients from two Brazilian oncology centers underwent targeted amplicon sequencing for the most prevalent *MUTYH* GPVs in Brazil. Comprehensive *MUTYH* sequencing was subsequently performed for monoallelic carriers. Overall, 25 (11.4%) patients carried at least one *MUTYH* GPV; among these, 15 (6.8%) were biallelic and classified as MAP and 10 (4.5%) were monoallelic. The MAP detection rate was 10.9% in patients <60 years. Compared with non‐carriers, MAP patients had an earlier CRC onset (49 vs. 59 years, *p* = 0.008), a higher prevalence of polyps (OR = 5.26; CI 95% 1.49–18.59; *p* = 0.036) and a family history of cancers (84.6% vs. 48.9%, *p* = 0.014), but fewer occurrences of metastasis (30.7% vs. 68.3%, *p* = 0.006) and stage IV tumors (30.8% vs. 68.3%, *p* = 0.029). Notably, most MAP cases (11/15) were not previously diagnosed, demonstrating that the strong association between *KRAS*‐G12C mutations and the presence of *MUTYH* GPVs supports its use as a biomarker for referring patients to germline *MUTYH* testing, enabling appropriate follow‐up, surveillance and preventive strategies for individuals at risk.

## Graphical abstract

What's new?

*MUTYH*‐associated polyposis is an underdiagnosed recessive syndrome that predisposes individual to colorectal cancer and frequently displays *KRAS*‐G12C mutations. By analyzing a large cohort of colorectal cancer patients routinely tested for *KRAS*, the authors assessed whether *KRAS*‐G12C detection could serve as a pre‐screening tool for the syndrome. Approximately 11% of the patients with *KRAS*‐G12C mutations carried at least one germline pathogenic variant in *MUTYH*, with 6.8% of these patients being biallelic and diagnosed with *MUTYH*‐associated polyposis. The findings support the use of *KRAS*‐G12C as a biomarker for referring patients to germline *MUTYH* testing, enabling appropriate follow‐up and surveillance strategies for these patients.

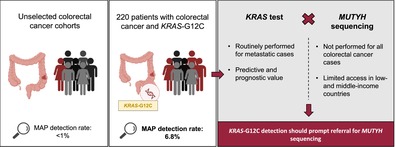

**Abbreviations**
ACC: A.C.Camargo Cancer Center
AS‐PCR: allele‐specific PCR
BCH: Barretos Cancer Hospital
CRC: colorectal cancer
FFPE: formalin‐fixed paraffin‐embedded
GPV: germline pathogenic variants
HM: homozygous
HT: heterozygous
LOH: loss of heterozygosity
MAP: *MUTYH*‐associated polyposis
NGS: next‐generation sequencing
PCR: polymerase chain reaction
SBS: single base substitution
VAF: variant allele frequency
yo: years old

## 1 INTRODUCTION

*MUTYH*‐associated polyposis (MAP) is a rare, autosomal recessive syndrome that significantly increases the lifetime risk of colorectal cancer (CRC) in both males and females. MAP accounts for 0.3%–0.7% of all CRCs and 7–13% of colorectal polyposis cases.^1^, ^2^ Considered an attenuated polyposis, patients typically present with 10 to a few hundred colonic polyps; however, some MAP patients develop CRC in the absence of polyposis and with limited family history, resulting in a highly variable phenotype that complicates diagnosis.^3^ The *MUTYH* gene plays a role in the DNA base excision repair pathway, repairing 8‐oxo‐guanines mispaired with adenines. This process is essential to prevent 8‐oxoG‐induced mutagenesis, a common oxidative DNA lesion that, if unrepaired, may cause G:C > T:A transversion mutations in colorectal cancer driver genes, such as *APC* and *KRAS*, and contribute to carcinogenesis.^4^ *MUTYH* biallelic inactivation leads to the accumulation of such mutations throughout the genome,^3^, ^5^ generating two specific mutational single base substitution (SBS) signatures SBS18 and SBS36^6^ (https://cancer.sanger.ac.uk/signatures/).

These transversions frequently lead to mutations in the *KRAS* oncogene and other crucial tumor suppressor genes in the intestinal mucosa.^5^ The most common transversion is the change in nucleotide 34 of *KRAS*, resulting in the p.G12C gain‐of‐function somatic mutation. While *KRAS*‐G12C occurs in <3% of sporadic CRC, it is found in over 80% of MAP patients^1^, ^2^, ^7^ Therefore, identifying *KRAS*‐G12C mutations could aid in diagnosing MAP patients. Previous studies analyzing 10 to 190 CRC patients with the *KRAS*‐G12C mutation found that 5%–25% had biallelic germline pathogenic variants (GPV) in *MUTYH*, suggesting that detecting this mutation could serve as a pre‐screening test for MAP.^1^, ^2^, ^7^, ^8^, ^9^ However, despite these findings, a widespread adoption of this biomarker has not been implemented.

In this study, we aimed to assess the utility of *KRAS*‐G12C detection in clinical practice as a biomarker for identifying carriers of GPVs in the *MUTYH* gene. We performed complete and/or partial sequencing of *MUTYH* in a Brazilian cohort of CRC patients harboring *KRAS*‐G12C mutations. Our findings support using *KRAS*‐G12C as a promising biomarker for identifying individuals with MAP.

## 2 MATERIALS AND METHODS

### 2.1 Study cohort

CRC patients over 18 years of age with *KRAS*‐G12C somatic mutations were selected from internal databases of the Genomic Diagnostic Laboratory at the A.C.Camargo Cancer Center (ACC) and the Laboratory of Molecular Diagnostic of Barretos Cancer Hospital (BCH) in Brazil. These databases included routine molecular tests (Pyrosequencing, Sanger sequencing, and Next‐Generation Sequencing [NGS]) for *KRAS* hotspots conducted between 2011 and 2022. We assessed the availability of germline DNA in institutional Biobanks and residual tumor DNA from formalin‐fixed paraffin‐embedded (FFPE) samples in diagnostic biorepositories. Patients with GPVs received results via genetic counseling at ACC and BCH.

Clinical and histopathological data were retrieved from electronic medical records and stored anonymously in REDCap. The term “presence of polyposis” was used when the patient's total polyp burden detected in all performed colonoscopies was at least 10; if the patient had fewer than 10 polyps detected, “presence of polyps” was used. Additional files can be found in the Data S1.

### 2.2 Multiplex polymerase chain reaction (PCR) and amplicon sequencing

DNA was extracted from leukocytes, saliva, or FFPE tissue following standardized protocols performed at the ACC and BCH Biobanks or diagnostic laboratories. A multiplex amplicon NGS method was used to screen for the five most common Brazilian *MUTYH* GPVs (p.Tyr179Cys, p.Gly396Asp, p.Arg241Trp, p.Ala385fs, and the deletion of exons 4–16). These five variants represent the most frequently reported in the literature among Brazilian patients^10^ and were identified in 12 out of 14 (85.7%), and in more than one, MAP patient followed by the ACC Oncogenetics department. Primers were designed with Primer3 software using primers designed with Primer 3 software (Table S1).

Multiplex PCR reactions using the Qiagen Multiplex PCR kit (Qiagen) were conducted with 20 ng of leukocyte or tumor DNA, following the manufacturer's instructions. The amplified products were used to build amplicon libraries with the Ion Plus Fragment Library kit and sequenced on the Ion S5 equipment, following the manufacturer's guidelines (Thermo Fisher Scientific). Sequencing reads were analyzed using Torrent Suite and Variant Caller software (Thermo Fisher Scientific) and compared to the GRCh37/Hg19 genome version. Variants detected in at least 30% of the total reads were considered positive and were visually inspected using the Integrative Genomics Viewer software.^11^

### 2.3 Complete sequencing of the MUTYH gene

Patients with only one heterozygous variant detected in the multiplex screening test were further evaluated by complete sequencing of the *MUTYH* gene using NGS multigene panels. For samples with leukocyte DNA available, a capture‐based panel evaluating 26 genes (Hereditary Cancer Solution—Sophia Genetics) was utilized, followed by sequencing on the NextSeq 500 platform (Illumina) and analysis using the Sophia DDM software (Sophia Genetics). For FFPE DNA samples, an amplicon‐based panel (Comprehensive Cancer Panel—Thermo Fisher Scientific) comprising 409 oncogenes and cancer‐related tumor suppressor genes was employed. A complete list of the genes targeted by each panel can be found in Tables S2 and S3. Libraries were prepared using the Ion AmpliSeq™ Library Kit 2.0 reagents, following the Ion AmpliSeq Library Preparation protocol (Thermo Fisher Scientific). Sequencing was carried out on the Ion S5 platform, with variant calling performed in the Torrent Suite software and variant annotation and filtering executed using VarSeq software (Golden Helix). Additional details on sequencing methods can be found in the Data S1 (Supplementary Materials and Methods). The sequencing coverage and quality statistics for each sample are summarized in Tables S4 and S5.

### 2.4 Allele‐specific PCR (AS‐PCR)

AS‐PCR was employed to confirm the phase of variants present in compound heterozygous individuals, determining whether the variants were located on the same or different chromosomes, as previously reported.^10^ This method was carried out only for compound heterozygous individuals with leukocyte or saliva DNA available, as it requires high molecular weight DNA. The PCR conditions matched those described for the multiplex PCR. Primers are available on Table S6.

### 2.5 Second hit analysis in monoallelic patients

Patients classified as monoallelic were evaluated for loss of heterozygosity (LOH) in tumor DNA, using an amplicon‐based PCR. The PCR conditions were the same as previously described. For patients confirmed as monoallelic in tumor samples, the LOH status was assessed from the 409‐gene panel data. LOH was confirmed if the tumor sample showed a Variant Allele Frequency (VAF) >65% of the total reads.^12^

### 2.6 Statistical Analysis

The clinical and histopathological characteristics of the patients were described using descriptive statistics. Student's *t*‐test was employed to compare continuous variables between two groups, while ANOVA and Tukey's test were applied for comparisons among more than two groups. Associations for categorical data were assessed using Fisher's exact test or Pearson's chi‐square test. A two‐tailed *p*‐value of <0.05 was considered statistically significant.

## 3 RESULTS

### 3.1 Detection of MUTYH GPVs in patients with CRC and KRAS‐G12C

**FIGURE 1:**
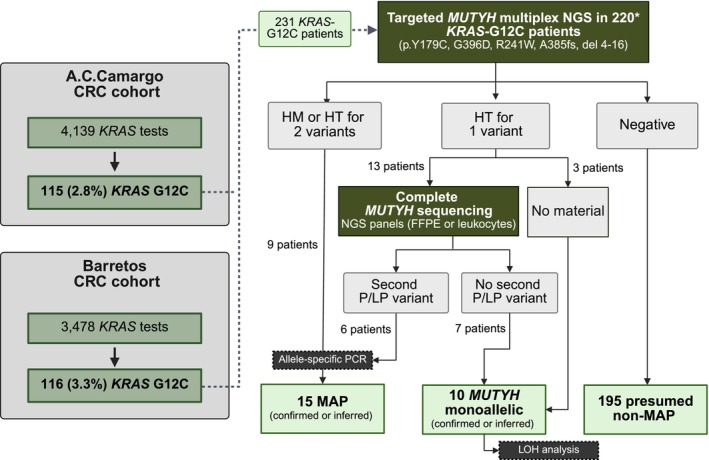
Flowchart summarizing the process of identifying germline pathogenic variants (GPV) in *KRAS*‐G12C patients. From a total of 7617 *KRAS* tests performed at ACC and BCH, 220 *KRAS*‐G12C patients were identified and underwent *MUTYH* multiplex NGS of the 5 most common Brazilian GPVs. These patients had three possible outcomes: (1) Homozygous or compound heterozygous patients were classified as “Confirmed” or “Inferred” MAP cases (9 patients); (2) Heterozygous patients carrying one of the evaluated variants underwent *MUTYH* complete sequencing (13 patients). When a second GPV was identified, the workflow for homozygous/compound heterozygous cases was applied (6 patients); if no additional variant was identified, the patient was classified as “Confirmed monoallelic” (7 patients). Patients with insufficient material for complete *MUTYH* sequencing were classified as “Inferred monoallelic” (3 patients). Monoallelic cases were then subjected to Loss of Heterozigosity (LOH) analysis. Finally, negative cases were classified as “Presumed non‐MAP”. This workflow identified 15 (6.8%) MAP patients, 10 (4.5%) *MUTYH* monoallelics and 195 (88.6%) negative patients. *MUTYH multiplex NGS was performed in 220/231 patients; 11 lacked DNA or tumor materials for analysis. CRC, colorectal cancer; HM, homozygous; HT, heterozygous; FFPE, formalin‐fixed paraffin‐embedded; LP, likely pathogenic; LOH, loss of heterozygosity; MAP, *MUTYH*‐associated polyposis; NGS, next‐generation sequencing; P, pathogenic; PCR, polymerase chain reaction.

From 7617 *KRAS* tests performed at ACC (*n* = 4139) and BCH (*n* = 3487), 231 (3%) CRC patients with tumors harboring the *KRAS* c.34G>T; p.G12C mutation were initially identified. Eleven patients lacked material for analysis, resulting in a final cohort of 220 patients included in the study (Figure 1 and Table S7). After multiplex sequencing, 25 (11.4%) carried at least one GPV in *MUTYH*, with 15 (6.8%) harboring biallelic GPVs, classified as MAP, and 10 (4.5%) harboring only one heterozygous GPV, classified as monoallelic. Among patients under 60 years old, 19/110 (17.2%) carried at least one GPV in *MUTYH*, with 12 (10.9%) classified as MAP and 7 (6.3%) as monoallelic (Tables 1 and 2). All targeted variants were identified at least once, with p.Tyr179Cys being the most frequent (40%), followed by p.Gly396Asp (28%), p.Ala385fs (20%), and deletion of exons 4–16 and p.Arg241Trp, each occurring in 12% of cases. Strikingly, only four (26.6%) MAP patients (ID10, ID21, ID22 and ID24) had been previously diagnosed as MAP through referral to the Oncogenetics service: one of eight cases from ACC and three of seven from BCH.

**TABLE 1:** *MUTYH* germline pathogenic variants detected in patients with CRC and *KRAS*‐G12C.

ID	Sex	Race	Age	Polyps	CRC family history	GPVs multiplex	GPVs *MUTYH* sequencing	Classification	Previous diagnosis of MAP
ID1	Female	White	58	Multiple	Absent	c.536A>G; p.Tyr179Cys^HT^	c.325C>G; p.Arg109Gly^HT^	Confirmed biallelic (MAP)	No
ID2	Male	Brown	27	1	Father (61yo), 2 paternal uncles	c.348 + 33_*64 + 146del4285; p.? (Del E4‐E16^HM^)	Not applicable	Confirmed biallelic (MAP)	No
ID3	Female	No data	56	Multiple	Absent	c.536A>G; p.Tyr179Cys^HT^/c.721C>T; p.Arg241Trp^HT^	Not applicable	Confirmed biallelic (MAP)	No
ID5	Female	White	44	2	Brother	c.348 + 33_*64 + 146del4285; p.? (Del E4‐E16^HM^)	Not applicable	Confirmed biallelic (MAP)	No
ID8	Male	White	50	33	Absent	c.1187G>A; p.Gly396Asp^HT^	c.933 + 3A>C; p.?^HT^	Confirmed biallelic (MAP)	No
ID14	Female	White	44	Multiple	Absent	c.348 + 33_*64 + 146del4285; p.? (Del E4‐E16^HT^)	c.688C>T; p.Gln230*^HT^	Confirmed biallelic (MAP)	No
ID23	Male	Brown	50	Absent	2 brothers (44yo and 49yo)	c.1147del; p.Ala385fs^HM^	Absent	Confirmed biallelic (MAP)	No
ID24	Male	Brown	68	Multiple	Absent	c.536A>G; p.Tyr179Cys^HM^	Absent	Confirmed biallelic (MAP)	Yes
ID25	Male	White	49	1	Absent	c.1187G>A; p.Gly396Asp^HM^	Not applicable	Confirmed biallelic (MAP)	No
ID4	Male	No data	63	No data	No data	c.1187G>A; p.Gly396Asp^HT^ / c.721C>T; p.Arg241Trp^HT^	Not applicable	Inferred biallelic (MAP)	No
ID7	Female	White	59	Absent	Aunt	c.536A>G; p.Tyr179Cys^HT^ / c.1187G>A; p.Gly396Asp^HT^	Not applicable	Inferred biallelic (MAP)	No
ID10	Female	White	32	1–10	Paternal great‐grand father (>60yo)	c.536A>G; p.Tyr179Cys^HT^	c.902C>G; p.Pro301Arg^HT^	Inferred biallelic (MAP)	Yes
ID15	Female	Brown	No data	No data	No data	c.536A>G; p.Tyr179Cys^HT^ / c.1147del; p.Ala385fs^HT^	Not applicable	Inferred biallelic (MAP)	No
ID21	Male	White	43	>50	Absent	c.1147del; p.Ala385fs^HT^	c.389‐1G>C; p. spl?^HT^	Inferred biallelic (MAP)	Yes
ID22	Female	Brown	43	Multiple	2 maternal cousins (45yo and 50yo)	c.1227_1228dup; p.Glu410fs^HT^ / c.1012C>T; p.Gln338*^HT^	Not applicable	Inferred biallelic (MAP)	Yes
ID6	Female	Brown	51	11	Absent	c.536A>G; p.Tyr179Cys^HT^	Absent	Confirmed monoallelic	–
ID9	Male	Brown	53	Multiple	Absent	c.536A>G; p.Tyr179Cys^HT^	Absent	Confirmed monoallelic	–
ID11	Male	White	64	2	Absent	c.1147del; p.Ala385fs^HT^	Absent	Confirmed monoallelic	–
ID12	Male	White	54	4	Absent	c.721C>T; p.Arg241Trp^HT^	Absent	Confirmed monoallelic	–
ID17	Male	Brown	44	No data	No data	c.1147del; p.Ala385fs^HT^	Absent	Confirmed monoallelic	–
ID18	Male	White	No data	1	No data	c.1187G>A; p.Gly396Asp^HT^	Absent	Confirmed monoallelic	–
ID19	Female	White	55	7	Absent	c.536A>G; p.Tyr179Cys^HT^	Absent	Confirmed monoallelic	–
ID13	Male	White	56	2	Absent	c.1187G>A; p.Gly396Asp^HT^	Could not be evaluated	Inferred monoallelic	‐
ID16	Female	White	31	Absent	Absent	c.1187G>A; p.Gly396Asp^HT^	Could not be evaluated	Inferred monoallelic	–
ID20	Male	White	75	4	Absent	c.536A>G; p.Tyr179Cys^HT^	Could not be evaluated	Inferred monoallelic	–

*Note*: Variants were described based on the transcript NM_001128425.2.

Abbreviations: CRC, Colorectal Cancer; ID, Identification; GPV, Germline pathogenic variant; HM, Homozygous; HT, Heterozygous; MAP, *MUTYH*‐associated polyposis; yo, Years old.

**TABLE 2:** Statistical analysis for clinical and histopathological characteristics comparing MAP patients vs. patients without GPVs in *MUTYH.*

Clinical and histopathological characteristics	Biallelic (MAP)	Without GPVs in *MUTYH*	*p*
	*N* (%)	*N* (%)	
Mean age (range)	49 (27–68)	59 (24–92)	0.008
Sex			
Female	8 (53.33%)	97 (50.52%)	0.834
Male	7 (46.67%)	95 (49.48%)	
Unknown	–	3	
Family history of CRC			
Present	6 (46.15%)	34 (25%)	0.100
Absent	7 (53.85%)	102 (75%)	
Unknown	2	59	
Family history of other cancers			
Present	11 (84.62%)	66 (48.89%)	0.014
Absent	2 (15.38%)	69 (51.11%)	
Unknown	2	60	
Polyps			
Present	11 (84.62%)	77 (54.61%)	0.036
Absent	2 (15.38%)	64 (45.39%)	
Unknown	2	54	
Polyposis			
Present	7 (53.85%)	9 (6.38%)	0
Absent	6 (46.15%)	132 (93.62%)	
Unknown	2	54	
Metastasis			
Present	4 (30.77%)	112 (68.29%)	0.006
Absent	9 (69.23%)	52 (31.71%)	
Unknown	2	31	
Other primary tumors			
Present	2 (15.38%)	26 (17.11%)	0.874
Absent	11 (84.62%)	126 (82.89%)	
Unknown	2	43	
Clinical stage			
I	0	4 (2.48%)	0.029
II	3 (23.08%)	14 (8.7%)	
III	6 (46.15%)	33 (20.5%)	
IV	4 (30.77%)	110 (68.32%)	
Unknown	2	34	
Tumor location			
Right	6 (42.86%)	50 (30.12%)	0.569
Left	8 (57.14%)	113 (68.07%)	
Appendix	0	3 (1.81%)	
Unknown	1	29	
Differentiation grade			
Well‐differentiated0	0	16 (11.11%)	0.317
Moderately differentiated	10 (83.33%)	119 (82.64%)	
Poorly differentiated	2 (16.67%)	9 (6.25%)	
Unknown	3	51	

Abbreviations: CRC, colorectal cancer; GPV, germline pathogenic variant; N, number of patients.

Of the 15 MAP patients, nine were considered “Confirmed MAP”, comprising five (ID2, ID5, ID23, ID24, ID25) homozygous cases and four (ID1, ID3, ID8, ID14) compound heterozygous cases (three confirmed by AS‐PCR and one [ID14] confirmed due to the presence of the 4–16 deletion in one allele and a nonsense variant in the non‐deleted allele). Six patients (ID4, ID7, ID10, ID15, ID21, ID22) who were double heterozygous cases but lacked phase confirmation, were considered “Inferred MAP”. Finally, seven patients (ID6, ID9, ID11, ID12, ID17, ID18, ID19) harboring only one heterozygous variant after complete *MUTYH* sequencing were considered “Confirmed Monoallelic”, while three others, for whom complete *MUTYH* sequencing could not be performed due to sample unavailability, were considered “Inferred Monoallelic” (Table 1).

### 3.2 Clinical and histopathological features

Among the 15 MAP patients, the age at CRC diagnosis ranged from 27 to 68 years. Of the 13/15 MAP patients with available polyp data, seven (63.6%) exhibited a polyposis phenotype. Most patients were White (61.5%), while sex and family history for CRC were evenly distributed, with no clear prevalence. Finally, only two cases had an additional primary tumor diagnosed during follow‐up: one lung carcinoma and one renal angiomyolipoma (Table 2).

Clinical data (mean age, sex, family history of CRC and other cancers, presence of polyps, and metastasis) and histopathological data (clinical stage, tumor location, and differentiation grade) were compared between patients with *MUTYH* biallelic variants and those without GPVs in *MUTYH* (Table 2). MAP patients had an earlier mean age of CRC onset (49 vs. 59, *p* = 0.008), a higher prevalence of polyps (84.62% vs. 54.61%, *p* = 0.036), polyposis (53.85% vs. 6.38%, *p* < 0.0001), and occurrence of other cancers in their families (84.62% vs. 48.89%, *p* = 0.014) compared to CRC patients without GPVs in MUTYH (Tables 2 and S8). Additionally, MAP patients exhibited a lower prevalence of metastasis and stage IV cancers (30.77% vs. 68.29%, *p* = 0.006 and 30.77% vs. 68.32%, *p* = 0.029, respectively).

**FIGURE 2:**
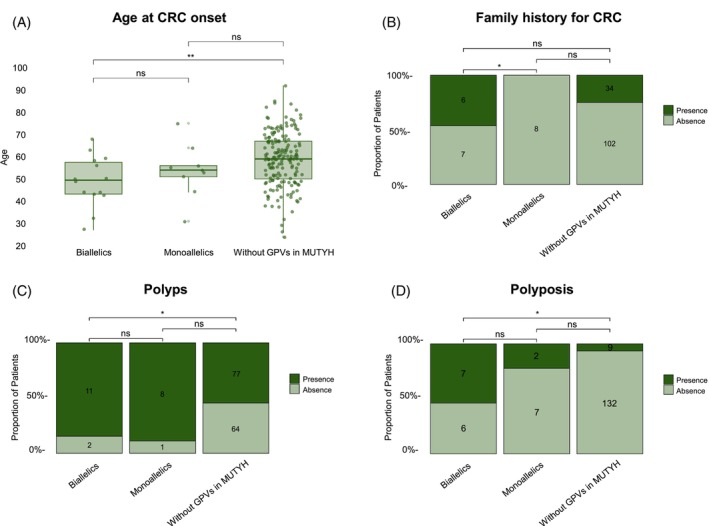
Comparison between biallelic, monoallelic, and patients without GPVs in *MUTYH* regarding mean age, family history of CRC, and the proportions of polyp and polyposis presence. (A) Box plot represents the age distribution of patients in the cohort, including means and standard deviations. (B) Proportions of patients with and without family history of CRC. (C) Proportions of patients with and without polyps (any number). (D) Proportions of patients with and without polyposis (≥10). *, Significant (*p* value <0.05); GPV, Germline pathogenic variant; NS, Not significant (*p*‐value >0.05).

Monoallelic patients do not differ from MAP patients or from those without GPVs in terms of age (53 vs. 49, *p* = 0.637 and 53 vs. 59, *p* = 0.394, respectively), polyps (8 vs. 11, *p* = 1 and 8 vs. 77, *p* = 0.078), and polyposis (2 vs. 7, *p* = 0.203 and 2 vs. 9, *p* = 0.132) (Figure 2A,C,D). However, monoallelic carriers showed a lower prevalence of family history of CRC when compared to biallelic patients (0 vs. 6, *p* = 0.045) (Figure 2B).

### 3.3 Analysis of second hits in monoallelic patients

Ten patients (ID6, ID9, ID11, ID12, ID13, ID16, ID17, ID18, ID19, ID20) confirmed or inferred as monoallelic underwent LOH analysis (Table 3). Four patients (ID6, ID11, ID17, ID20) exhibited a VAF >65%, confirming LOH of the wild allele, while six (ID9, ID12, ID13, ID16, ID18, ID19) did not present LOH. One patient (ID19) showed a borderline VAF (64%) and was considered inconclusive.

**TABLE 3:** Variants detected in *MUTYH* complete sequencing and loss of heterozygosity (LOH) analysis in monoallelic patients.

ID	Variant	GPV VAF	Tumor cellularity	*KRAS*‐G12C VAF
ID6	c.536A>G; p.Tyr179Cys	88% (LOH)	70%	No data
ID9	c.536A>G; p.Tyr179Cys	42%	90%	No data
ID11	c.1147del; p.Ala385fs	67% (LOH)	No data	No data
ID12	c.721C>T; p.Arg241Trp	45%	90%	No data
ID13	c.1187G>A; p.Gly396Asp	48%	No data	No data
ID16	c.1187G>A; p.Gly396Asp	54%	No data	29%
ID17	c.1147del; p.Ala385fs	81% (LOH)	No data	28%
ID18	c.1187G>A; p.Gly396Asp	51%	No data	25%
ID19	c.536A>G; p.Tyr179Cys	64%	No data	45%
ID20	c.536A>G; p.Tyr179Cys	67% (LOH)	No data	24%

*Note*: Variants were described based on the transcript NM_001128425.2.

Abbreviations: ID, Identification; GPV, Germline pathogenic variant; VAF, variant allele frequency.

## 4 DISCUSSION

In this study, we systematically investigated the association between the *KRAS*‐G12C somatic mutation and biallelic *MUTYH* germline alterations in Brazilian CRC patients. By analyzing a large cohort of CRC patients routinely tested for *KRAS*, we aimed to determine whether the presence of *KRAS*‐G12C could serve as a molecular clue prompting germline testing for MAP. Our findings demonstrate that referring these patients for germline testing would lead to high detection rates of *MUTYH* GPVs (11.4%) and MAP cases (6.8%), particularly in those under 60 years old (10.9%). These detection rates were notably higher than those in CRC cohorts (0.3%–0.7%) and the general population (0.01%).^1^, ^2^

Previous studies have also reported a high frequency of MAP patients in *KRAS*‐G12C CRC cohorts. Yanus et al.^8^ identified 6 MAP patients (6.7%) in a cohort of 90 *KRAS*‐G12C tumors, and Georgeson et al.^2^ detected 16 (11%) out of 143 *KRAS*‐G12C/CRC cases as MAP. In contrast, Aimé et al.^1^ identified a higher frequency (7/28–25%) than those from previous findings. However, this data may be biased due to the small cohort size and missing clinical data especially on age and polyps. A Russian study by Volkov et al.,^9^ in turn, reported a lower frequency (4.73%) of MAP in *KRAS*‐G12C CRC cases, which may be due to a higher frequency of *KRAS*‐G12C mutations in the Russian population compared to others and/or the existence of other common GPVs in the Russian population that were not included in their initial screening (Table 4).

**TABLE 4:** Comparison between the present study and previous studies assessing MAP detection in CRC cohorts with somatic *KRAS*‐G12C mutation.

Study	Number of CRC with *KRAS*‐G12C mutation	Number of *MUTYH* biallelic (MAP) (%)
Aimé et al. 2015^1^	28	7 (25%)
Yanus et al. 2018^7^	90	6 (6.7%)
Volkov et al. 2020^8^	190	9 (4.73%)
Georgeson et al. 2022^2^	143	16 (11%)
This study	220	15 (6.8%)

*Note*: CRC, Colorectal cancer; MAP, *MUTYH*‐associated polyposis.

We showed that MAP patients exhibited an earlier CRC onset (*p* = 0.008) and a higher incidence of polyps (*p* = 0.036) compared to patients without GPVs in *MUTYH*. Additionally, *MUTYH* biallelic patients had more non‐CRC cases in their families (*p* = 0.014), which remain challenging to interpret since genetic testing was not performed on family members. MAP patients also showed a lower prevalence of stage IV tumors (*p* = 0.029) and metastasis (*p* = 0.006), perhaps as a result of earlier/more frequent screening due to the history of polyps or the recently suggested higher immunogenicity of MAP tumors.^2^, ^13^, ^14^ Nevertheless, further studies are needed to explore these findings.

We identified 4.5% of monoallelic patients in our cohort, a higher frequency than described for unselected colorectal cases (1.5–2.1%)^2^, ^15^ or in a healthy Brazilian population (1.96%).^16^ The association of monoallelic *MUTYH* variants with cancer risk, clinical impact, and management remains controversial. Current guidelines do not recommend specific screening unless there is a personal or first‐degree family history of CRC or polyps.^17^ While some studies found no increased risk for certain cancers,^15^, ^18^, ^19^ others suggest a low to moderate risk for tumors such as gastric adenocarcinomas, adrenal adenocarcinomas and pancreatic neuroendocrine tumors.^14^, ^20^, ^21^ The proposed mechanisms for cancer development in monoallelic individuals include second somatic hits (point mutations), variants not covered by common panels, synergistic effects with GPVs in other CRC‐related genes, and LOH of the wild‐type allele in the tumor, which is the most widely demonstrated mechanism.^14^, ^22^, ^23^, ^24^ In our *KRAS*‐G12C cohort, four of 10 (40%) monoallelic patients exhibited LOH, a greater value than previous reports (4%–12%) with unselected CRC cohorts.^2^, ^14^

Our study has some limitations. Due to the limited availability of biological samples, some biallelic and monoallelic *MUTYH* carriers were considered inferred rather than confirmed. Despite screening for the five most common variants in the Brazilian population, it is possible that some affected patients were missed due to GPVs occurring in other regions. Finally, since most *KRAS*‐G12C patients in our study had advanced disease stages (stages III and IV), as predictive CRC targeted panels are typically conducted for metastatic cases,^8^, ^25^ the true frequency of MAP in all CRC stages may be underestimated.

Genetic testing for *MUTYH* and other polyposis‐associated genes is typically recommended for individuals with more than 10 adenomas; however, there are no clear guidelines for *MUTYH* testing that consider the variable polyp burden in MAP.^17^ Although MAP is classified as an attenuated polyposis, about one‐third of patients present with isolated CRC or fewer than 10 polyps.^26^, ^27^ In our cohort, 46.1% (6/13) lacked a polyposis phenotype. Importantly, only four of the 15 MAP patients, all of whom presented with polyposis, had undergone prior genetic testing, highlighting the underdiagnosis of MAP in the routine clinical setting.

While KRAS somatic analysis is routinely performed in CRC using targeted NGS panels or comprehensive genomic profiles due to its prognostic and predictive relevance for KRAS‐G12C targeted therapies,^28^, ^29^ the use of KRAS‐G12C as a MAP biomarker remains neglected. Recently, the detection of SBS18 and SBS36 in tumors has also been postulated as a MAP indicator.^2^, ^30^ However, despite their high sensitivity and specificity, their assessment often relies on whole‐exome or whole‐genome sequencing, which is less accessible in clinical routine. Moreover, even considering the National Comprehensive Cancer guidelines' suggestion for considering genetic testing for all diagnosed CRC cases, this approach remains largely unimplemented in most low‐ and middle‐income countries.^31^ Therefore, incorporating *KRAS*‐G12C as a biomarker could improve MAP diagnosis, enabling regular surveillance, preventive measures, and reducing CRC incidence among patients and their families.

In summary, our findings reveal an increased prevalence of biallelic *MUTYH* carriers among CRC patients with the *KRAS*‐G12C somatic mutation, underscoring that its detection should prompt *MUTYH* genetic testing, even in the absence of a family history of CRC or a polyposis condition. This mutation is routinely tested in clinical practice and can help identify individuals with MAP syndrome beyond traditional phenotype‐based approaches, especially in less‐resourced regions.

## AUTHOR CONTRIBUTIONS

**Ana Beatriz Deleame Medeiros:** Formal analysis; investigation; methodology; writing – original draft; writing – review and editing. **Gabriel Oliveira dos Santos:** Formal analysis; resources; writing – review and editing. **José Claudio Casali‐da‐Rocha:** Resources; writing – review and editing. **Samuel Aguiar Junior:** Resources; writing – review and editing. **Virgilio Souza Silva:** Resources; writing – review and editing. **Gustavo Nóriz Berardinelli:** Formal analysis; resources; writing – review and editing. **Augusto Perazzolo Antoniazzi:** Resources; writing – review and editing. **Rui Manuel Reis:** Funding acquisition; resources; supervision; writing – review and editing. **Dirce Maria Carraro:** Funding acquisition; supervision; resources; writing – review and editing. **Giovana Tardin Torrezan:** Conceptualization; investigation; formal analysis; funding acquisition; supervision; resources; writing – review and editing; writing – original draft.

## FUNDING INFORMATION

This study was supported by the Collaborative Group of the Americas on Inherited Gastrointestinal Cancer [2022 CGA‐IGC Research Grant]; the São Paulo Research Foundation [FAPESP 2014/50943‐1, 2022/05162‐8 and 2023/01303‐9]; Barretos Cancer Hospital, Barretos; Public Ministry of Labor Campinas (Research, Prevention, and Education of Occupational Cancer), Campinas, Brazil; the National Council for Scientific and Technological Development [CNPq: 465682/2014‐6]; and the Coordination for the Improvement of Higher Education Personnel [CAPES: 88887.136405/2017‐00].

## CONFLICT OF INTEREST STATEMENT

Giovana Tardin Torrezan has received grants from Servier and has received honoraria for lectures from Roche. Virgilio Souza e Silva has received honoraria for lectures from MSD, Amgen, Servier, Pfizer, Merck; has received support for attending meetings and/or travel from Pfizer, MSD, Merck, Amgen, Merck and has participated on advisory boards for Pfizer, MSD. All remaining authors have declared no conflicts of interest.

## ETHICS STATEMENT

This study was approved by the institutional ethics review boards (CAAE 53067621.3.0000.5432 and 53067621.3.3001.5437). Patients who were included in the study had signed informed consent, either through the institutional Biobank or specifically for this project.

## Supporting information


**TABLE S1.** Variants, primers, and amplicon sizes for the MUTYH multiplex PCR.
**TABLE S2.** List of 26 genes assessed by the Hereditary Cancer Solution (Sophia Genetics).
**TABLE S3.** List of 409 genes assessed by the Comprehensive Cancer Panel (Thermo Fisher Scientific).
**TABLE S4.** Sequencing statistics of leukocyte samples processed on the MiniSeq platform (Illumina).
**TABLE S5.**. Sequencing statistics of FFPE samples processed on the Ion S5 platform (Thermo Fisher Scientific).
**TABLE S6.** Variants, primers, and amplicon sizes for the AS PCR.
**TABLE S7.** Clinical and histopathological characteristics of the 220 KRAS‐G12C patients.
**TABLE S8.** Types of non‐colorectal cancers occurred in family members of MAP patients.

## Data Availability

The data that support the findings of this study are available from the corresponding author upon reasonable request.
