# Supplementary material for: KRAS ‐G12C: The neglected biomarker to detect patients with MUTYH ‐associated polyposis
Source: Int J Cancer. 2025 Dec 5;158(6):1588–98. doi: 10.1002/ijc.70281 (PMC12811217; doi:10.1002/ijc.70281)
Supplement: Supplementary file 1 — TABLE S1. Variants, primers, and amplicon sizes for the MUTYH multiplex PCR. TABLE S2. List of 26 genes assessed by the Hereditary Cancer Solution (Sophia Genetics). TABLE S3. List of 409 genes assessed by the Comprehensive Cancer Panel (Thermo Fisher Scientific). TABLE S4. Sequencing statistics of leukocyte samples processed on the MiniSeq platform (Illumina). TABLE S5.. Sequencing statistics of FFPE samples processed on the Ion S5 platform (Thermo Fisher Scientific). TABLE S6. Variants, primers, and amplicon sizes for the AS PCR. TABLE S7. Clinical and histopathological characteristics of the 220 KRAS‐G12C patients. TABLE S8. Types of non‐colorectal cancers occurred in family members of MAP patients. [file IJC-158-1588-s001.pdf]

KRAS-G12C: The neglected biomarker to detect patients with *MUTYH*-associated polyposis

Authors: Ana Beatriz Deleame Medeiros, Gabriel Oliveira dos Santos, José Claudio Casali-da-Rocha, Samuel Aguiar Junior, Virgilio Souza e Silva, Gustavo Nóriz Berardinelli, Augusto Perazzolo Antoniazzi, Rui Manuel Reis, Dirce Maria Carraro, Giovana Tardin Torrezan.

Supplementary Material – Table of Contents

Supplementary Materials and Methods 1. Complete sequencing of the *MUTYH* gene on NGS panels 2

Supplementary Table 1. Variants, primers, and amplicon sizes for the *MUTYH* multiplex PCR. 3

Supplementary Table 2. List of 26 genes assessed by the Hereditary Cancer Solution (Sophia Genetics). 3

Supplementary Table 3. List of 409 genes assessed by the Comprehensive Cancer Panel (Thermo Fisher Scientific). 4

Supplementary Table 4. Sequencing statistics of leukocyte samples processed on the MiniSeq platform (Illumina). 5

Supplementary Table 5. Sequencing statistics of FFPE samples processed on the Ion S5 platform (Thermo Fisher Scientific). 5

Supplementary Table 6. Variants, primers, and amplicon sizes for the AS-PCR. .. 5

Supplementary Table 7. Clinical and histopathological characteristics of the 220 *KRAS*-G12C patients. 6

Supplementary Table 8. Types of non-colorectal cancers occurred in family members of MAP patients. 7

Supplementary Materials and Methods 1. Complete sequencing of the *MUTYH* gene on NGS panels

For samples with leukocyte DNA available, a capture-based panel evaluating 26 genes (Hereditary Cancer Solution - Sophia Genetics) was utilized, followed by paired-end 150 read sequencing with a MiniSeq High Output Kit on the MiniSeq platform (Illumina). Analyses were performed using the Sophia DDM software (Sophia Genetics), with standard quality control, variant calling and filtering protocols validated by the Genomic Diagnostic Laboratory of A.C.Camargo Cancer Center.

For FFPE DNA samples, an amplicon-based panel (Comprehensive Cancer Panel - Thermo Fisher Scientific) comprising 409 oncogenes and cancer-related tumor suppressor genes was employed. Libraries were prepared using the Ion AmpliSeq™ Library Kit 2.0 reagents, following the Ion AmpliSeq Library Preparation protocol (Thermo Fisher Scientific). Sequencing was carried out with Ion 540™ Chip Kit on the Ion S5 platform, for a total of 500 cycles. Variant calling and coverage analysis were performed in the Torrent Suite software plugins variantCaller v5.18 and coverageAnalysis v5.18, respectively. Standard quality control, variant calling and filtering protocols were validated by the Genomic Diagnostic Laboratory of A.C.Camargo Cancer Center. Final variant annotation and filtering specific for *MUTYH* gene was executed using VarSeq software (Golden Helix). Variants were considered true when variant allele frequencies were higher 5%.

Supplementary Table 1. Variants, primers, and amplicon sizes for the *MUTYH* multiplex PCR.

Variant	Primer forward	Primer reverse	Amplicon size (bp)
c.1187G>A; p.Gly396Asp (exon 13)	5’GGCAGTGGCATGAGTAACAA3’	5’GACGGGAACTCCCACAGTC3’	112
c.536A>G; p.Tyr179Cys (exon 7)	5’ACCCACAGGAGGTGAATCAA3’	5’CCAAGACTCCTGGGTTCCTA3’	120
c.721C>T; p.Arg241Trp (exon 9)	5’GGTGTGGTGGATGGCAAC3’	5’CCCAACATCCTACCAGAGCT3’	105
c.1147del; p.Ala385fs (exon 12)	5’AGCTCTGCCACCTGTGTTCT3’	5’GCCGATTCCCTCCATTCTCT3’	140
Exons 4-16 deletion	5’CAGAACCAAGCAGAACGTCA3’	5’GCCTGCTAAGCTGGTACGAC3’	188

Note: bp - Base pairs. *MUTYH* transcript - NM_001128425.2.

Supplementary Table 2. List of 26 genes assessed by the Hereditary Cancer Solution (Sophia Genetics).

ATM	*APC*	*BARD1*	*BRCA1*
BRCA2	*BRIP1*	*CDH1*	*CHEK2*
EPCAM	*FAM175A*	*MLH1*	*MRE11A*
MSH2	*MSH6*	*MUTYH*	*NBN*
PALB2	*PIK3CA*	*PMS2*	*PTEN*
RAD50	*RAD51c*	*RAD51d*	*STK11*
TP53	*XRCC2*		

Supplementary Table 3. List of 409 genes assessed by the Comprehensive Cancer Panel (Thermo Fisher Scientific).

ABL1	*ABL2*	*ACVR2A*	*ADAMTS20*	*AFF1*	*AFF3*	*AKAP9*
AKT1	*AKT2*	*AKT3*	*ALK*	*APC*	*AR*	*ARID1A*
ARID2	*ARNT*	*ASXL1*	*ATF1*	*ATM*	*ATR*	*ATRX*
AURKA	*AURKB*	*AURKC*	*AXL*	*BAI3*	*BAP1*	*BCL10*
BCL11A	*BCL11B*	*BCL2*	*BCL2L1*	*BCL2L2*	*BCL3*	*BCL6*
BCL9	*BCR*	*BIRC2*	*BIRC3*	*BIRC5*	*BLM*	*BLNK*
BMPR1A	*BRAF*	*BRD3*	*BTK*	*BUB1B*	*CARD11*	*CASC5*
CBL	*CCND1*	*CCND2*	*CCNE1*	*CD79A*	*CD79B*	*CDC73*
CDH1	*CDH11*	*CDH2*	*CDH20*	*CDH5*	*CDK12*	*CDK4*
CDK6	*CDK8*	*CDKN2A*	*CDKN2B*	*CDKN2C*	*CEBPA*	*CHEK1*
CHEK2	*CIC*	*CKS1B*	*CMPK1*	*COL1A1*	*CRBN*	*CREB1*
CREBBP	*CRKL*	*CRTC1*	*CSF1R*	*CSMD3*	*CTNNA1*	*CTNNB1*
CYLD	*CYP2C19*	*CYP2D6*	*DAXX*	*DCC*	*DDB2*	*DDIT3*
DDR2	*DEK*	*DICER1*	*DNMT3A*	*DPYD*	*DST*	*EGFR*
EML4	*EP300*	*EP400*	*EPHA3*	*EPHA7*	*EPHB1*	*EPHB4*
EPHB6	*ERBB2*	*ERBB3*	*ERBB4*	*ERCC1*	*ERCC2*	*ERCC3*
ERCC4	*ERCC5*	*ERG*	*ESR1*	*ETS1*	*ETV1*	*ETV4*
EXT1	*EXT2*	*EZH2*	*FAM123B*	*FANCA*	*FANCC*	*FANCD2*
FANCF	*FANCG*	*FANCJ*	*FAS*	*FBXW7*	*FGFR1*	*FGFR2*
FGFR3	*FGFR4*	*FH*	*FLCN*	*FLI1*	*FLT1*	*FLT3*
FLT4	*FN1*	*FOXL2*	*FOXO1*	*FOXO3*	*FOXP1*	*FOXP4*
FZR1	*G6PD*	*GATA1*	*GATA2*	*GATA3*	*GDNF*	*GNA11*
GNAQ	*GNAS*	*GPR124*	*GRM8*	*GUCY1A2*	*HCAR1*	*HFN1A*
HIF1A	*HLF*	*HOOK3*	*HRAS*	*HSP90AA1*	*HSP90AB1*	*ICK*
IDH1	*IDH2*	*IGF1R*	*IGF2*	*IGF2R*	*IKBKB*	*IKBKE*
IKZF1	*IL2*	*IL21R*	*IL6ST*	*IL7R*	*ING4*	*IRF4*
IRS2	*ITGA10*	*ITGA9*	*ITGB2*	*ITGB3*	*JAK1*	*JAK2*
JAK3	*JUN*	*KAT6A*	*KAT6B*	*KDM5C*	*KDM6A*	*KEAP1*
KIT	*KLF6*	*KOR*	*KRAS*	*LAMP1*	*LCK*	*LIFR*
LPHN3	*LPP*	*LRP1B*	*LTF*	*LTK*	*MAF*	*MAFB*
MAGEA1	*MAGI1*	*MALT1*	*MAML2*	*MAP2K1*	*MAP2K2*	*MAP2K4*
MAP3K7	*MAPK1*	*MAPK8*	*MARK1*	*MARK4*	*MBD1*	*MCL1*
MDM2	*MDM4*	*MEN1*	*MET*	*MITF*	*MLH1*	*MLL*
MLL2	*MLL3*	*MLLT10*	*MMP2*	*MN1*	*MPL*	*MRE11A*
MSH2	*MSH6*	*MTOR*	*MTR*	*MTRR*	*MUC1*	*MUTYH*
MYB	*MYC*	*MYCL1*	*MYCN*	*MYD88*	*MYH11*	*MYH9*
NBN	*NCOA1*	*NCOA2*	*NCOA4*	*NF1*	*NF2*	*NFE2L2*
NFKB1	*NFKB2*	*NIN*	*NKX2-1*	*NLRP1*	*NOTCH1*	*NOTCH2*
NOTCH4	*NPM1*	*NRAS*	*NSD1*	*NTRK1*	*NTRK3*	*NUMA1*
NUP214	*NUP98*	*PAK3*	*PALB2*	*PARP1*	*PAX3*	*PAX5*
PAX7	*PAX8*	*PBRM1*	*PBX1*	*PDE4DIP*	*PDGFB*	*PDGFRA*
PDGFRB	*PER1*	*PGAP3*	*PHOX2B*	*PIK3C2B*	*PIK3CA*	*PIK3CB*
PIK3CD	*PIK3CG*	*PIK3R1*	*PIK3R2*	*PIM1*	*PKHD1*	*PLAG1*
PLCG1	*PLEKHG5*	*PML*	*PMS1*	*PMS2*	*POT1*	*POU5F1*
PPARG	*PPP2R1A*	*PRDM1*	*PRKAR1A*	*PRKDC*	*PSIP1*	*PTCH1*
PTEN	*PTGS2*	*PTPN11*	*PTPRD*	*PTPRT*	*RADSO*	*RAF1*
RALGDS	*RARA*	*RB1*	*RECQL4*	*REL*	*RET*	*RHOH*
RNASEL	*RNF2*	*RNF213*	*ROS1*	*RPS6KA2*	*RRM1*	*RUNX1*
RUNX1T1	*SAMD9*	*SBDS*	*SDHA*	*SDHB*	*SDHC*	*SEPT9*
SETD2	*SF3B1*	*SGK1*	*SH2D1A*	*SMAD2*	*SMAD4*	*SMARCA4*
SMARCB1	*SMO*	*SMUG1*	*SOCS1*	*SOHD*	*SOX11*	*SOX2*
SRC	*SSX1*	*STK11*	*STK36*	*SUFU*	*SYK*	*SYNE1*
TAF1	*TAF1L*	*TAL1*	*TBX22*	*TCF12*	*TCF3*	*TCF7L1*
TCF7L2	*TCL1A*	*TET1*	*TET2*	*TFE3*	*TGFBR2*	*TGM7*
THBS1	*TIMP3*	*TLR4*	*TLX1*	*TNFAIP3*	*TNFRSF14*	*TNK2*
TOP1	*TP53*	*TPR*	*TRIM24*	*TRIM33*	*TRIP11*	*TRRAP*
TSC1	*TSC2*	*TSHR*	*UBR5*	*UGT1A1*	*USP9X*	*VHL*
WAS	*WHSC1*	*WRN*	*WT1*	*XPA*	*XPC*	*XPO1*
XRCC2	*ZNF384*	*ZNF521*				

Supplementary Table 4. Sequencing statistics of leukocyte samples processed on the MiniSeq platform (Illumina).

Sample ID	Total number of sequenced reads	Total number of uniquely mapped reads	Total number of covered targeted bases	Median coverage per targeted base*	Coverage homogeneity	Percentage of target bases with coverage >200x
ID3	883,166	869,957	104,776	438	100%	99.81%
ID9	1,007,566	992,754	104,776	508	99.99%	99.93%
ID11	3,561,710	3,503,956	104,776	1244	99.96%	99.98%

Note: ID – Identification. Genome assembly GRCh37(hg19).

Supplementary Table 5. Sequencing statistics of FFPE samples processed on the Ion S5 platform (Thermo Fisher Scientific).

Sample ID	Total number of sequenced reads	Total number of uniquely mapped reads	Total number of covered targeted bases	Median coverage per targeted base*	Uniformity of base coverage	Percentage of target bases with coverage >100x	Percentage of target bases with coverage >500x
ID2	24,364,451	24,307,174	1,688,650	1,496	87.36%	94.75%	78.52%
ID4**	-	>20,000,00	1,688,650	-	>85%	>95%	-
ID6	41,133,011	40,765,822	1,688,650	2,348	86.03%	96.26%	85.16%
ID14	19,354,909	19,233,925	1,688,650	1,149	86.77%	94.26%	67.13%
ID17	38,533,786	38,204,667	1,688,650	2,239	86.91%	95.97%	85.25%
ID18	49,307,405	49,112,402	1,688,650	3,070	86.11%	96.77%	88.93%
ID19	21,701,136	21,587,754	1,688,650	1,325	82.96%	93.86%	67.07%
ID21	45,618,681	45,329,467	1,688,650	2,651	79.39%	95.29%	80.62%
ID23	35,282,194	34,988,415	1,688,650	1,999	87.82%	96.37%	83.62%
ID24	53,678,908	53,350,531	1,688,650	3,157	87.94%	96.77%	90.51%

Note: ID – Identification. **ID4 sample was sequenced on a facility service and detailed data on coverage was no longer available. Genome assembly GRCh37(hg19).

Supplementary Table 6. Variants, primers, and amplicon sizes for the AS-PCR.

Variant 1	Variant 2	ASP 1	ASP 2	CON	Amplicon size (bp)
c.325C>G; p.Arg109Gly (exon 7)	c.536A>G; p.Tyr179Cys (exon 7)	5'CTGGGCTGG CCTGGGCTA3' (reverse)	5'CTGGGCTGG CCTGGGCTG3' (reverse)	5'GCCTGCTAA GCTGGTACGAC3' (forward)	663
c.721C>T; p.Arg241Trp (exon 9)	c.536A>G; p.Tyr179Cys (exon 7)	5'CTGGGCTGG CCTGGGCTA3' (reverse)	5'CTGGGCTGG CCTGGGCTG3' (reverse)	5'CACCCTTGT TACCCCAACAT3' (forward)	455
c.1187G>A; p.Gly396Asp (exon 13)	c.933+3A>C; p. spl? (intron 10)	5'CACAGTCCT GCCAGCAGAC3' (reverse)	5'CACAGTCCT GCCAGCAGAT3' (reverse)	5'CACAGTGTG TACCCCACAGC3' (forward)	701

Note: bp - Base pairs. ASP – Primer allele-specific. CON – Conserved primer. *MUTYH* transcript - NM_001128425.2.

Supplementary Table 7. Clinical and histopathological characteristics of the 220 *KRAS*-G12C patients.

Clinical and Histopathological characteristics		Number of patients (%)
**Mean age (range)**		58 (24-92)
**Sex**	Female	108 (49.77%)
	Male	109 (50.23%)
	Unknown	3
**Family history of CRC**	Present	40 (25.48%)
	Absent	117 (74.52%)
	Unknown	63
**Family history of other cancers**	Present	81 (51.92%)
	Absent	75 (48.08%)
	Unknown	64
**Polyps**	Present	96 (58.9%)
	Absent	67 (41.1%)
	Unknown	57
**Polyposis**	Present	18 (11.04%)
	Absent	145 (88.96%)
	Unknown	57
**Metastasis**	Present	123 (65.78%)
	Absent	64 (34.22%)
	Unknown	33
**Other primary tumors**	Present	29 (16.67%)
	Absent	145 (83.33%)
	Unknown	46
**Clinical stage**	I	4 (2.17%)
	II	18 (9.78%)
	III	41 (22.28%)
	IV	121 (65.76%)
	Unknown	36
**Tumor location**	Right	59 (31.05%)
	Left	128 (67.37%)
	Appendix	3 (1.58%)
	Unknown	30
**Differentiation grade**	Well-differentiated	18 (11.04%)
	Moderately-differentiated	134 (82.21%)
	Poorly-differentiated	11 (6.75%)
	Unknown	57

Note: CRC – Colorectal cancer.

Supplementary Table 8. Types of non-colorectal cancers occurred in family members of MAP patients.

Patient	Cancer type	Family member
ID1	Cervical[1], central nervous system[2]	Sister[1], sister[2]
ID3	Leukemia	Maternal aunt
ID5	Stomach[1], larynx[2], central nervous system [3]	Mother, brother[1], father[2], brother[3]
ID7	Throat	Grandmother
ID8	Throat	Paternal uncle
ID10	Liver	Paternal grandmother
ID14	Kidney	Sister
ID21	Lung[1], throat[2]	Mother[1], maternal aunt[2]
ID22	Stomach[1], esophagus[2]	Paternal aunt[1], paternal aunt[2]
ID23	Leukemia	Brother
ID25	Uterus, breast	Sister

Note: ID – Identification. Cancer type was described as how it was reported in the clinical record.

* The range of the Median coverage is unavailable, however, we included in both tables other metrics that serve to the same purpose of attesting the uniformity and minimal coverage of regions: "Uniformity of base coverage" and "Percentage of target bases with coverage >100x or 200x".
